# Protein kinase Cδ mediates histamine-evoked itch and responses in pruriceptors

**DOI:** 10.1186/1744-8069-11-1

**Published:** 2015-01-06

**Authors:** Manouela V Valtcheva, Steve Davidson, Chengshui Zhao, Michael Leitges, Robert W Gereau

**Affiliations:** Washington University Pain Center and Department of Anesthesiology, Washington University in St. Louis, 660 S. Euclid Ave, Box 8054, 63110 St. Louis, MO USA; Medical Scientist Training Program, Washington University in St. Louis, St. Louis, MO 63110 USA; Biotechnology Centre of Oslo, University of Oslo, Blindern, N-0317 Oslo, Norway

**Keywords:** PKC isoform, PKCdelta, Novel PKC, Peptide inhibitor, Pruritus

## Abstract

**Background:**

Itch-producing compounds stimulate receptors expressed on small diameter fibers that innervate the skin. Many of the currently known pruritogen receptors are G_q_ Protein-Coupled Receptors (G_q_PCR), which activate Protein Kinase C (PKC). Specific isoforms of PKC have been previously shown to perform selective functions; however, the roles of PKC isoforms in regulating itch remain unclear. In this study, we investigated the novel PKC isoform PKCδ as an intracellular modulator of itch signaling in response to histamine and the non-histaminergic pruritogens chloroquine and β-alanine.

**Results:**

Behavioral experiments indicate that PKCδ knock-out (KO) mice have a 40% reduction in histamine-induced scratching when compared to their wild type littermates. On the other hand, there were no differences between the two groups in scratching induced by the MRGPR agonists chloroquine or β-alanine. PKCδ was present in small diameter dorsal root ganglion (DRG) neurons. Of PKCδ-expressing neurons, 55% also stained for the non-peptidergic marker IB4, while a smaller percentage (15%) expressed the peptidergic marker CGRP. Twenty-nine percent of PKCδ-expressing neurons also expressed TRPV1. Calcium imaging studies of acutely dissociated DRG neurons from PKCδ-KO mice show a 40% reduction in the total number of neurons responsive to histamine. In contrast, there was no difference in the number of capsaicin-responsive neurons between KO and WT animals. Acute pharmacological inhibition of PKCδ with an isoform-specific peptide inhibitor (δV1-1) also significantly reduced the number of histamine-responsive sensory neurons.

**Conclusions:**

Our findings indicate that PKCδ plays a role in mediating histamine-induced itch, but may be dispensable for chloroquine- and β-alanine-induced itch.

## Background

Itch, clinically known as pruritus, is an unpleasant sensory and emotional experience that leads to the desire to scratch [1, 2]. Chronic itch can result in severe anxiety, self-mutilation, and impaired overall quality of life that is comparable to chronic pain [3–5]. Several histamine-dependent and histamine-independent itch receptors have been recently identified; however, few of the intracellular mediators downstream of these receptors have been characterized. Elucidating the intracellular mediators that activate pruriceptors may provide a new set of targets to aid in the generation of more specific and efficacious treatments.

Intradermal histamine induces itch via direct activation of the H_1_ histamine receptor, which is expressed in sensory neurons [6–10]. Additionally, several subtypes of the recently characterized class of Mas-related G protein-coupled receptors (MRGPR) have been shown to respond selectively to a variety of non-histaminergic, itch-producing compounds. For example, MRGPRA3 is activated by the anti-malarial drug chloroquine (CQ) [11, 12], and β-alanine induces itch by activating a subset of nonpeptidergic MRGPRD-expressing sensory neurons [13, 14].

A common property of many of the identified pruritogen receptors, including the H_1_ histamine receptor (H_1_R), MRGPRA3, and MRGPRD, is that they are G_q_ protein-coupled receptors (G_q_PCRs) [10–12, 14]. Canonically, G_q_PCRs activate phospholipase C (PLC), which cleaves phosphatidylinositol (PIP_2_) into inositoltriphosphate (IP_3_) and diacylglycerol (DAG), resulting in release of intracellular calcium stores and activation of downstream targets. However, the itch-mediating factors downstream of PLC are largely unknown [10, 15]. Protein kinase C (PKC) is coupled to the canonical G_q_PCR/PLC pathway via activation by DAG and/or calcium and therefore may play a role in the signaling of itch.

A number of PKC isoforms are expressed in sensory neurons [16–19]. One of these isoforms is PKCδ, a member of the “novel” PKC isozymes, which depends on DAG but not calcium for its activation. Previously, we demonstrated that PKCδ is dispensable for withdrawal responses to acute noxious mechanical and thermal stimuli [20]. However, studies of H_1_R signaling in human aortic endothelial cells and HeLa cells showed that PKCδ is phosphorylated in response to histamine [21, 22]. PKCδ also mediated histamine-induced H_1_R mRNA upregulation and downstream activation of ERK1/2 and p38 [21, 22]. These lines of evidence suggest that PKCδ could play a specific role as an intracellular modulator of itch in sensory neurons.

In this study, we tested the hypothesis that PKCδ contributes to pruritogen-induced itch. We determined the role of PKCδ in histaminergic and non-histaminergic itch by examining scratching responses to histamine and the non-histaminergic pruritogens chloroquine and β-alanine, which activate separate pruriceptor subpopulations. We characterized the distribution of PKCδ in sensory neurons and show that both genetic deletion and pharmacological inhibition of PKCδ significantly decrease the proportion of histamine-responsive neurons.

## Results

### PKCδ mediates histamine-induced itch

**Figure 1:**
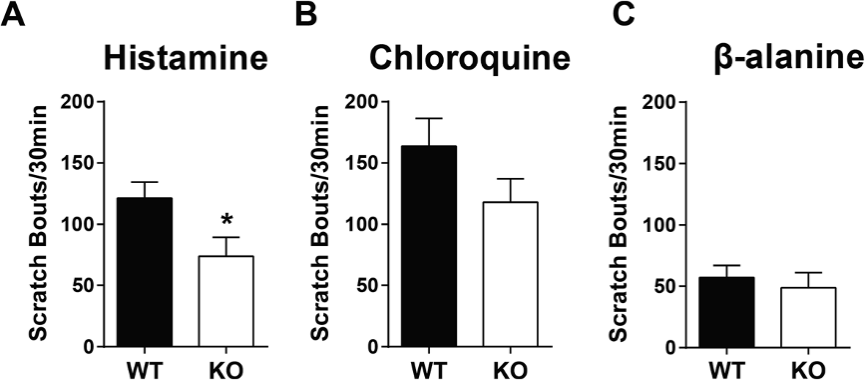
**PKCδ** **-KO and WT scratching responses to pruritogens. A**. PKCδ-KO mice scratched less than wild type littermates in response to intradermal histamine injection (WT = 121.4 ± 12.8 scratch bouts/30 min, n = 25; KO = 73.8 ± 15.5 scratch bouts/30 min, n = 19; unpaired t test p < 0.05). **B**. Chloroquine (CQ)-induced scratching was not different between PKCδ-KO and WT mice (WT = 163.7 ± 22.6 scratch bouts/30 min, n = 23; KO = 118.0 ± 18.9 scratch bouts/30 min, n = 21; unpaired t test p = 0.129). **C**. Scratch bouts induced by β-alanine were also not different between PKCδ-KO and WT mice (WT = 57.0 ± 10 scratch bouts/30 min, n = 6; KO = 48.8 ± 12.3 scratch bouts/30 min, n = 8; unpaired t test p = 0.61).

To determine if PKCδ plays a role in behavioral responses to itch, we assessed scratching responses to histamine and non-histaminergic pruritogens in PKCδ knock-out mice (PKCδ-KO) and their wild type littermates. Mice were injected intradermally at the nape of the neck with one of three pruritogens: histamine (1 mg), chloroquine (CQ) (200 μg), or β-alanine (223 μg). PKCδ-KO mice scratched significantly less than their wild type littermates when injected with histamine (Figure 1A, p < 0.05). On the other hand, chloroquine-induced scratching was not significantly different between WT and PKCδ KO mice (Figure 1B, p = 0.129). There was also no difference in the number of scratch bouts induced by β-alanine (Figure 1C, p = 0.61). These results indicate that PKCδ mediates histaminergic itch, but is not necessary for non-histaminergic itch induced by CQ and β-alanine.

### PKCδ is preferentially expressed in small diameter DRG neurons

**Figure 2:**
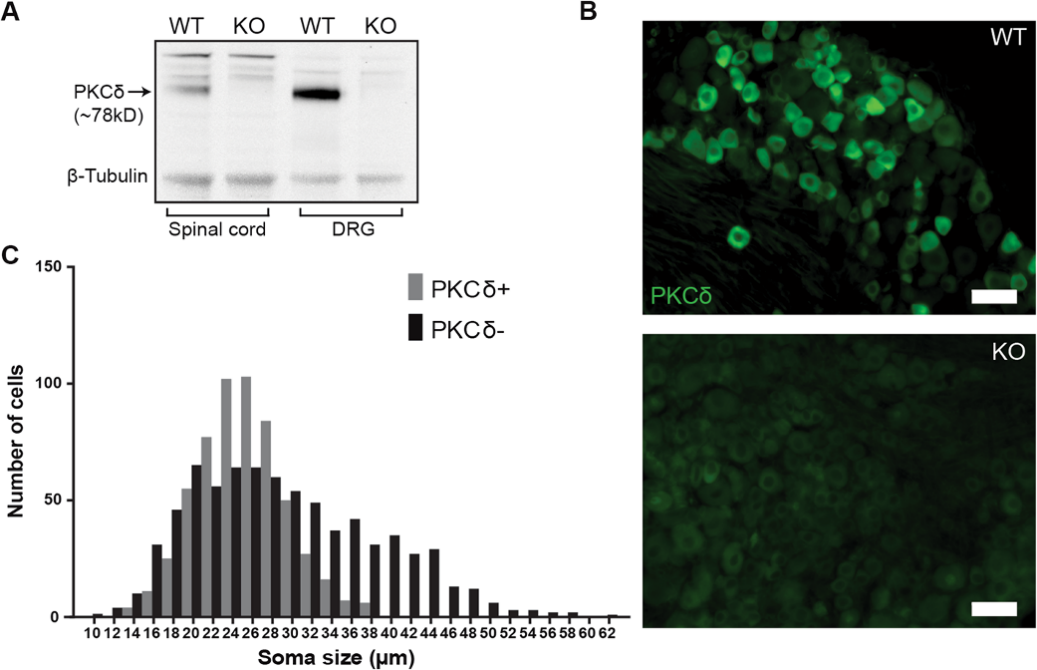
**Immunological analyses of PKCδ** **in the spinal cord and DRG. A**. Western blot demonstrating expression of PKCδ in WT DRG and spinal cord (SC) but not in PKCδ-KO tissue confirming the validity of the PKCδ antibody. **B**. Representative images of 18 μm sections from WT and PKCδ-KO lumbar DRG. (Scale bar = 50 μm) **C**. Histogram of cell diameter measurements of PKCδ+ and PKCδ- neurons illustrates the localization of PKCδ to small and medium diameter soma.

PKCδ is expressed in a variety of tissues, including the brain and peripheral nervous system [19, 21, 23–30]. To assess whether PKCδ is localized to potential pruriceptive sensory neurons, immunohistochemistry (IHC) was used to characterize the distribution of PKCδ in dorsal root ganglion (DRG) neurons. Antibody specificity was confirmed via western blot using PKCδ-KO DRG and spinal cord tissue. No antibody staining was found corresponding to the 78kD PKCδ band in PKCδ-KO DRG tissue (Figure 2A). This was further confirmed by IHC of knock-out and wild type DRG (Figure 2B). In wild type lumbar DRG, PKCδ was expressed in 43.2% of total neurons labeled with βIII tubulin (567/1314 cells, n = 3 animals) and PKCδ expression was predominantly restricted to small diameter neurons (average diameter 23.3 ± 0.19 μm, min 11.6 μm, max 36.2 μm) (Figure 2C).

### PKCδ is expressed in peptidergic and non-peptidergic DRG neurons

**Figure 3:**
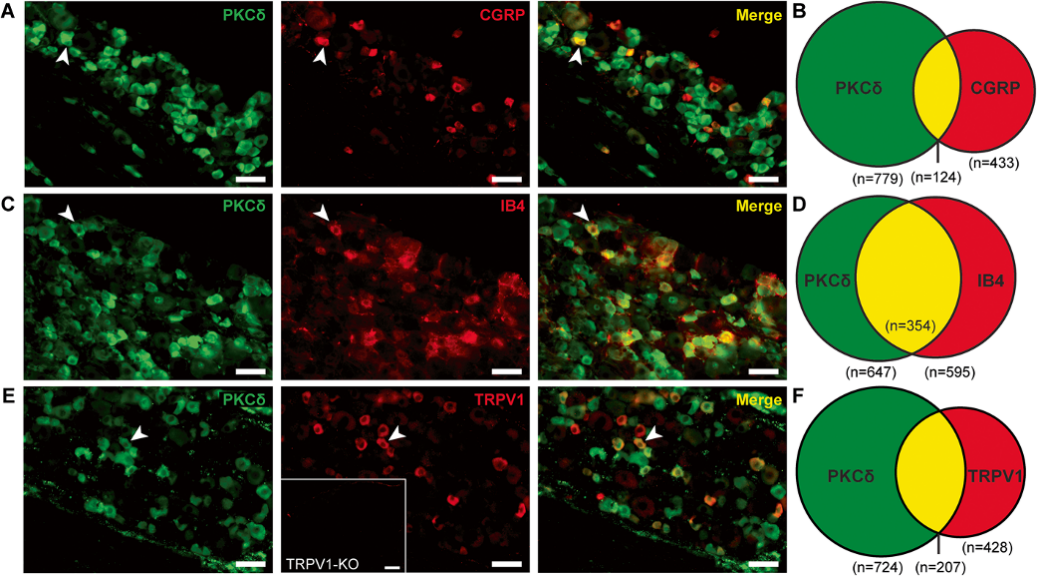
**Localization of PKCδ** **and markers of peptidergic and non-peptidergic dorsal root ganglion neurons. A**. Representative images of CGRP+, PKCδ+, and co-expressing (Merge) DRG neurons. **B**. Graphical representation of total neurons counted and degree of overlap (n = number of neurons). **C**, **D**. Representative images of IB4+, PKCδ+, and IB4+/PKCδ + neurons and illustration of overlap. **E**, **F**. Representative images of TRPV1+, PKCδ+, and TRPV+/PKCδ + neurons and illustration of overlap. Inset demonstrates TRPV1 antibody stain in TRPV1-KO mice. (Scale bar = 50 μm; Arrowheads indicate example cells that express both markers).

We further characterized PKCδ expression in small diameter DRG neurons by immunohistochemical analysis of peptidergic and non-peptidergic markers. Of peptidergic neurons identified by anti-Calcitonin Gene Related Peptide (CGRP+) immunoreactivity, 26.8% expressed PKCδ, while 14.7% of PKCδ-expressing neurons were CGRP-positive (Table 1, Figure 3A-B). PKCδ was also expressed in non-peptidergic neurons identified by isolectin B4 (IB4) binding. Of IB4+ DRG neurons, 61.6% expressed PKCδ and 55.0% of PKCδ+ neurons exhibited IB4 binding (Table 1, Figure 3C-D). These findings indicate that PKCδ is expressed in both peptidergic and non-peptidergic sensory neurons, with greater expression overlap found with non-peptidergic IB4+ neurons.

**Table 1:** **Percent of DRG neurons in which PKC**δ **colocalizes with other markers (mean ± SEM)**

Marker	% of PKCδ+ neurons expressing marker	% of marker+ neurons expressing PKCδ
**CGRP**	14.7 ± 2.5	26.8 ± 3.5
**IB4**	55.0 ± 3.0	61.6 ± 7.9
**TRPV1**	29.1 ± 2.2	48.9 ± 4.0

n = 3–4 mice per marker.

Behavioral and physiological studies have shown that itch produced by histamine is largely dependent on the non-specific cation channel transient receptor potential vanilloid receptor 1 (TRPV1) [31–33]. Because PKC has previously been demonstrated to modulate TRPV1 function and may present one potential mechanism by which PKCδ regulates itch, we determined the degree of overlap between PKCδ and TRPV1 expression [34, 35]. We first confirmed the specificity of our antibody directed against TRPV1 using TRPV1 knockout mice (Figure 3E, inset). Our data indicate that 29.1% of PKCδ-positive neurons were also TRPV1-positive, and 48.9% of TRPV1-positive neurons also expressed PKCδ, suggesting a potential functional relationship between PKCδ and TRPV1 (Figure 3E-F; Table 1).

### PKCδ-KO sensory neurons exhibit diminished responses to histamine

**Figure 4:**
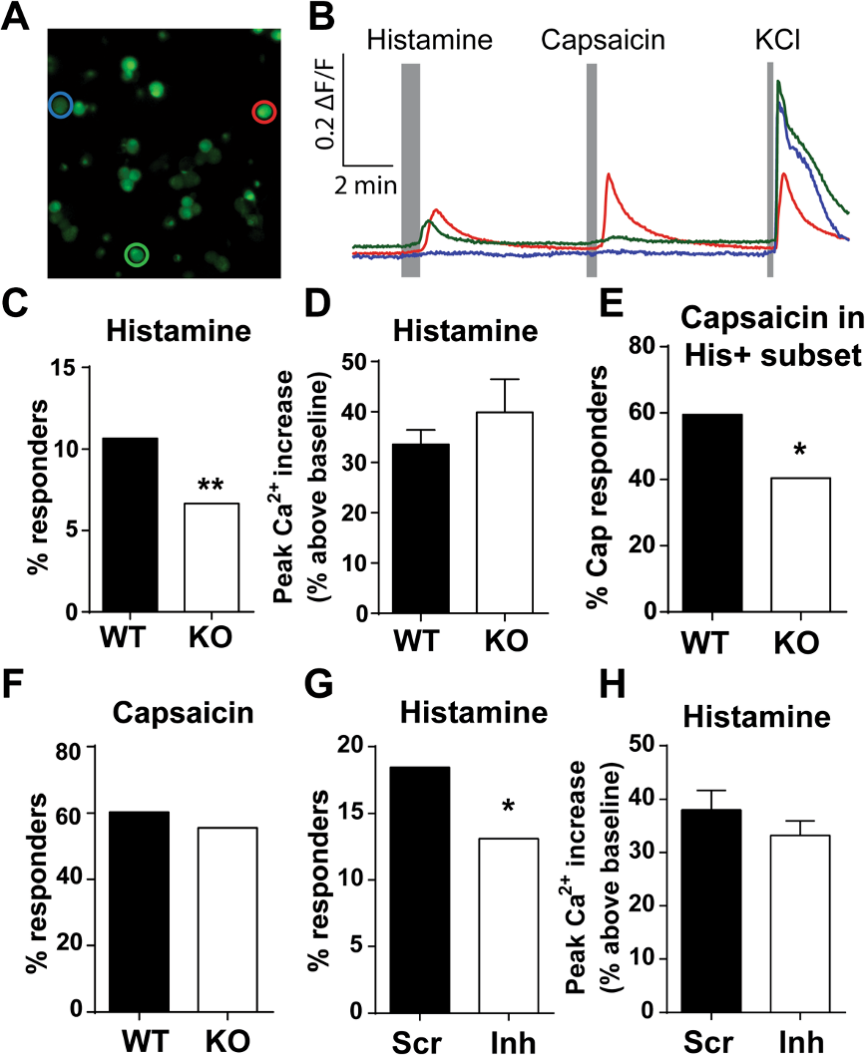
**PKCδ** **mediates sensory neuron responses to histamine. A**. Representative image of dissociated DRG neurons loaded with Fura-2 AM. **B**. Representative traces of selected cells (corresponding colored circles in **A** in response to histamine, capsaicin, and KCl. **C**. Proportion of total histamine responders in WT and KO DRG neurons (WT = 11.1% (126/1137 total WT neurons); KO = 6.7% (47/706 total KO neurons); **p < 0.01, χ^2^ test). **D**. Peak calcium increase in response to histamine, defined as % signal increase above baseline. WT 33.6 ± 2.8% change from baseline, n = 126 cells; KO 39.9 ± 6.6% change from baseline, n = 47 cells, unpaired t-test, p = 0.304. **E**. Percent of histamine-responsive neurons that responded to capsaicin (74/126 WT His + neurons; 19/47 KO His + neurons; p < 0.05, χ^2^ test). **F**. Proportion of capsaicin-responsive neurons (117/194 WT neurons, 144/259 KO neurons, p = 0.315, χ^2^ test). **G**. Proportion of histamine-responsive neurons in scramble- vs. inhibitor-treated groups (18.5% (75/405) of scramble-treated neurons vs. 13.1% (69/527) of peptide-treated neurons, p < 0.05, χ^2^ test). **H**. Peak calcium increase in response to histamine (inhibitor: 33.23 ± 2.7% change from baseline, n = 69 cells; scramble: 41.0 ± 3.7% change from baseline, n = 75 cells, p = 0.095, unpaired t test).

PKCδ is expressed in the brain, spinal cord, and the peripheral nervous system [30, 36–39]. Therefore, global genetic deletion of PKCδ in our knockout mice makes it difficult to pinpoint where PKCδ functions to modulate histamine-induced scratching. The expression of PKCδ in small diameter sensory neurons suggests that it may mediate histamine-evoked itch by signaling in nociceptive neurons responsive to pruritic agents (i.e., pruriceptors). To determine if PKCδ directly modulates neuronal responses to histamine, calcium imaging was performed on acutely dissociated adult mouse DRG neurons (Figure 4A-B). Of the total sensory neurons treated with histamine, 11.1% of wild type neurons responded to bath application of 100 μM histamine (126/1137 total WT neurons, N = 5 animals), but only 6.7% of PKCδ-KO neurons responded to histamine (47/706 total KO neurons, N = 3 animals), indicating a significant reduction of 39.6% in the proportion of histamine responsive neurons (p < 0.01, χ^2^ test) (Figure 4C). No significant difference in peak calcium responses to histamine was detected between knock-out and wild type cells (WT 33.6 ± 2.8% increase from baseline, n = 126 cells; KO 39.9 ± 6.6% increase from baseline, n = 47 cells, unpaired t-test, p = 0.304) (Figure 4D).

We hypothesized that PKCδ could contribute to neuronal responses to histamine by mediating histamine receptor coupling to TRPV1, or by regulating the normal expression or function of TRPV1. To investigate whether the absence of PKCδ affects the activation of TRPV1 within histamine-responsive neurons, we applied the TRPV1-specific agonist capsaicin after the histamine response. We found that 58.7% of WT histamine-responsive neurons subsequently responded to capsaicin (74/126 total His+ neurons), while only 40.4% of KO histamine-sensitive neurons responded to capsaicin (19/47 total His+ neurons; p < 0.05, χ^2^ test) (Figure 4E). To determine whether the functional expression of TRPV1 is altered in PKCδ-KO neurons, we tested WT and KO sensory neurons for responses to capsaicin. There were no differences in the total number of capsaicin-responsive neurons between KO and WT groups (117/194 (60.3%) WT neurons, 144/259 (55.6%) KO neurons, p = 0.315, χ^2^ test, Figure 4F). Together, these data indicate that the reduction in histamine responses is not due to altered levels of TRPV1 receptors in the KO, and support the idea that PKCδ could modulate TRPV1 downstream of histamine receptor activation.

To further control for the possibility of developmental effects or compensatory mechanisms that may occur with congenital genetic deletion of PKCδ, we performed calcium imaging experiments with the same experimental design using acute pharmacological inhibition in wild type DRG cultures. The δV1-1 peptide inhibitor has been shown to inhibit PKCδ activation *in vitro* and *in vivo* by competitively binding to receptor for activated C-kinase (RACK) proteins, which confer PKC isoform substrate specificity [28, 40–43]. DRG neurons were incubated with the peptide inhibitor δV1-1 for 30 minutes prior to recording. Consistent with our findings in PKCδ-KO neurons, there was a significant reduction in the total number of histamine-responsive neurons treated with peptide inhibitor when compared to scramble peptide-treated control neurons (18.5% (75/405) of scramble-treated neurons vs. 13.1% (69/527) of peptide-treated neurons, N = 4 animals, p < 0.05, χ^2^ test) (Figure 4G). Peak calcium responses induced by histamine were not different between inhibitor- and scramble-treated groups (inhibitor 33.23 ± 2.7% change from baseline, n = 69 cells; scramble 41.0 ± 3.7% change from baseline, n = 75 cells, p = 0.095, unpaired t test) (Figure 4H).

## Discussion

Pruritic stimuli can activate sensory neurons via specific intracellular signaling cascades, which represent potential targets for anti-pruritics, but these cascades remain poorly understood. Characterizing signaling components is a significant challenge, in part because of the great diversity of recently identified pruritic receptors. Some receptors involved in pruritus are coupled to G_i/o_ cascades such as the H4 histamine receptor, while others utilize kinase signaling pathways like the TSLP/IL-7 receptors [6, 7, 44, 45]. However, the majority of identified pruritic receptors, including the H_1_ receptor and the “orphan” family of MRGPR receptors, are linked by a common G_αq_ signaling mechanism [10, 46–49]. In this study we focused on a component downstream of the canonical G_αq_ signaling pathway, the serine/threonine kinase PKC.

PKC isozymes are divided into three groups: classic (activated by DAG and Ca^2+^), novel (activated by DAG but not Ca^2+^), and atypical (activated by neither DAG nor Ca^2+^) [50, 51]. Specific PKC isoforms have been shown to selectively regulate nociceptive behavior and nociceptor physiology [16, 17, 19, 52–58]. We previously showed that the novel isozyme PKCδ is dispensable for acute mechanical and thermal nociceptive behaviors [20]. Previous reports have implicated PKCδ in H_1_R signaling in non-neuronal cells [21, 22], but the specific role of PKCδ in pruriceptor signaling and itch had not been explored. In this study we found that PKCδ was necessary for the full expression of histamine-induced itch, but it did not have significant effects on histamine-independent itch produced by the MRGPR ligands chloroquine or β-alanine.

To determine whether the scratching deficit we observed in PKCδ null mice could be attributed to loss of function within sensory neurons, we examined anti-PKCδ staining in mouse lumbar DRG. Previous studies indicated that PKCδ is expressed in murine spinal cord and DRG, but the precise subset of PKCδ-positive neurons had not been characterized [30, 36–39]. We found that PKCδ expression was restricted to small diameter dorsal root ganglion neurons. Furthermore, although PKCδ was expressed in both peptidergic and non-peptidergic sensory neurons, it was greatly enriched in the non-peptidergic subset. Both peptidergic and nonpeptidergic fibers have been shown to play a role in pruritus. Histaminergic itch is largely dependent on CGRPα-positive neurons [59], however, a subset of histamine-responsive neurons also express the nonpeptidergic marker IB4 [11, 60].

Calcium imaging studies of dissociated DRG neurons demonstrated that genetic deletion of PKCδ resulted in a significant reduction of the proportion of adult sensory neurons that were histamine-responsive. We further confirmed these results using acute pharmacological inhibition, supporting the hypothesis that PKCδ functions within normal wild type sensory neurons to mediate acute histaminergic signaling. The expression of PKCδ in small diameter sensory neurons, along with the reduction of histamine-responsive sensory neurons, suggest a peripheral mechanism for the behavioral effects of global PKCδ deletion on histamine-induced scratching.

Following histamine release, sensory neuron signaling to produce itch is thought to depend on functional coupling of H_1_R to TRPV1. Supporting this idea, mice lacking TRPV1 exhibit greatly reduced scratching behavior and cellular responses to histamine, and blocking TRPV1 channel function likewise abolishes the response of sensory neurons to histamine [31, 33, 61]. The mechanisms by which H_1_R recruits TRPV1 are complex and several different signaling pathways have been implicated [32, 33, 62, 63]. One possible mechanism by which histamine could couple to TRPV1 in sensory neurons is through PLC-induced PKC activation. This is further supported by an expanding body of literature indicating that PKC directly modulates TRPV1 function [34, 35]. Indeed, inhibitors for PLC and PKC prevent histamine-induced TRPV1-potentiation [62]. We previously found that acute mechanical and thermal pain were independent of PKCδ. In contrast, PKCδ was necessary for the full expression of thermal hyperalgesia during Complete Freund’s Adjuvant-induced inflammation, which is a TRPV1 dependent process [20].

In this study, 49% of TRPV1-expressing neurons were PKCδ-positive and we showed a significant reduction in the proportion of capsaicin-responsive neurons within the subset of neurons responsive to histamine. Additionally, sensory neurons from PKCδ-KO mice not previously treated with histamine responded similarly to wild type neurons when challenged with capsaicin. These observations indicate that acute detection of heat stimuli by TRPV1 is not dependent on PKCδ, but suggest that PKCδ could function downstream of the histamine receptor to modulate TRPV1 function. In further support of this idea, several other inflammatory mediators including PGE_2_, NGF, and IL-6 have been shown to activate PKCδ [39, 64–66]. It is possible that pruritic dermatoses marked by inflammation may recruit PKCδ, resulting in sensory neuron modulation that could potentiate itch.

In contrast to histaminergic signaling, the MRGPR receptors appear to produce their pruritic effects through an alternative, PKCδ-independent pathway. Chloroquine activates MRGRPA3 which couples to the irritant receptor TRPA1 to produce itch. Neuronal responses to chloroquine were prevented by inhibiting Gβγ subunit activity, suggesting that the G_αq_ pathway is not necessary for chloroquine-induced itch [15]. However, MRGPRA3 was also recently shown to sensitize TRPV1 via a PKC mechanism likely dependent on G_αq_ signaling, suggesting a possible mechanism for thermal sensitization [67]. This suggests that MRGPRA3 may have biased signaling mechanisms that lead to itch and/or TRPV1-related sensitization. Thus, the histamine receptor and MRGPRs may share a pathway leading to sensitization of TRPV1 via PKC signaling, despite an alternative Gβγ mechanism for MRGPRA3 signaling of itch [46, 68].

In summary, we found that PKCδ is a mediator of histaminergic itch signaling in sensory neurons. Although we specifically investigated PKCδ in this study, other PKC isoforms may also be involved in modulating the response to itch. For example, another novel PKC isozyme, PKCϵ, is expressed in largely IB4+ neurons, and has been shown to also modulate TRPV1 responses [16, 57, 58, 69]. The sensory neuron responses involved in itch are complex, involving multiple molecular cascades which may be differentially mediated by specific PKC isoforms. Future studies that investigate the roles of PKC isozymes in itch may contribute to better therapeutic specificity for the treatment of acute and chronic pruritus.

## Conclusions

Our studies indicate that PKCδ significantly contributes to histamine-induced scratching behavior, but may be dispensable for non-histaminergic itch induced by the pruritogens chloroquine and β-alanine. In the peripheral nervous system, PKCδ expression is restricted to small diameter sensory neurons, and is found in both peptidergic and nonpeptidergic neurons. Physiological studies of cultured adult DRG demonstrate that PKCδ mediates histamine-induced responses of sensory neurons using genetic and pharmacological tools. In addition, PKCδ may act downstream of the histamine receptor to modulate TRPV1 activity. We conclude that PKCδ regulates sensory neuron responses necessary for acute histaminergic itch, and future studies should address the role of PKCδ in persistent and inflammatory pruritic conditions. Because PKCδ shows no effects on acute pain [20], but contributes to histaminergic itch, PKCδ inhibition may be a potential therapeutic target to selectively control pruritus.

## Methods

### Subjects and ethical approval

All experiments were conducted in accordance with the National Institute of Health guidelines and received the approval of the Animal Care and Use Committee of Washington University School of Medicine. 8–12 week old male littermate mice were housed on a 12 hour light–dark cycle and allowed ad libitum access to food and water.

PKCδ-KO mice were obtained from Dr. Michael Leitges [70]. These mice were generated using a standard gene targeting approach to insert a LacZ/neo cassette in the first transcribed exon of the PKCδ gene to abolish transcription, resulting in a global knock-out [70]. PKCδ-KO mice were backcrossed on a C57BL/6 background for at least 6 generations prior to use. PKCδ-KO mice were then crossed with wild type C57BL/6 mice to generate heterozygous mice, which were used to generate wild type and KO littermates.

### Pruritogen-induced scratching behavior

The nape of the neck and upper back were shaved with electric clippers one day prior to behavioral experiments. On the day of experiment, mice were placed in individual plexiglass observation boxes and allowed to acclimate in the presence of white noise for 2 hours. Using gentle restraint, 50 μl consisting of pruritogen dissolved in 0.9% normal saline was injected intradermally at the nape of the neck using a 29½ gauge insulin syringe. The following pruritogen amounts were used: 1 mg histamine (Sigma Aldrich, St. Louis, MO), 200 μg chloroquine (Sigma Aldrich, St. Louis, MO), and 223 μg β-alanine (Sigma Aldrich, St. Louis, MO). A single scratch bout was defined as one or more rapid back-and-forth motions of the hindpaw directed at the injection site, ending with either a pause, licking, or biting of the toes or placing of the hindpaw on the floor. Scratch bouts by the hind-paw directed at the injection site were counted over a period of 30 minutes. Experimenters were blinded to mouse genotype.

### PKCδ immunohistochemistry/Western Blotting protocol

For Western blotting, mice were euthanized by swift decapitation and lumbar spinal cord and lumbar DRG were removed. Tissue samples were homogenized in homogenization buffer (20 mM Tris–HCl, pH 7.4, 1 mM EDTA, 1 mM sodium pyrophosphate, 25 μg/ml aprotinin, 25 μg/ml leupeptin and 100 μM phenylmethylsufonyl fluoride) on ice. 7 μg of spinal cord and DRG protein were separated using 4-12% SDS-PAGE, then transferred to nitrocellulose membrane. Membrane was blocked in Odyssey blocking buffer for 1 hour, then incubated in rabbit anti-PKCδ (1:1000, Santa Cruz) and mouse anti-β-Tubulin (1:1000, Sigma-Aldrich) primary antibodies in Odyssey buffer with 0.1% Tween-20 at 4°C overnight. Blots were then washed in TBS-0.1% Tween-20, and incubated for 1 hour at room temperature in secondary antibodies (goat anti-rabbit Alexa Fluor 680 (1:20,000, Sigma Aldrich); goat anti-mouse IR800 (1:20,000, Sigma Aldrich)). Blots were washed and scanned using an Odyssey infrared scanner.

For immunohistochemistry (IHC), mice were deeply anesthetized with a ketamine, xylazine, and acepromazine cocktail, then perfused intracardially with cold PBS followed by 4% paraformaldehyde in PBS. Lumbar DRG were removed and cryoprotected in 30% sucrose. Transverse sections were cut at 18 μm thickness on a cryostat and collected on slides. To determine the percentage of total neurons that express PKCδ, dual labeling was performed with rabbit anti-PKCδ (1:50, Santa Cruz) and mouse anti-β-tubulin (1:1000, Sigma Aldrich) primary antibodies. Briefly, sections were blocked in 2% BSA, 0.1% Milk powder, 0.05% Tween-20 TBS for 1 hr, then incubated in primary antibodies overnight at 4°C. On day 2, slides were washed and incubated in secondary antibodies for 2–4 hours at 4°C (Alexa Fluor 488 Donkey anti-rabbit 1:200, Alexa Fluor 555 donkey anti-mouse 1:200, Invitrogen). Images were obtained using an upright epifluorescent microscope (Nikon 80i, CoolSnapES camera). Labeled neurons were counted in at least 3 randomly selected sections separated by >50 μm per animal. The size distribution of PKCδ+ neurons was determined using ImageJ software to measure cell diameter. The percentage of PKCδ+ neurons that also expressed CGRP or IB4 was determined using dual labeling for PKCδ and CGRP (goat anti-CGRP 1:400, Serotec) or Alexa-568 conjugated to IB4 (1:400, Invitrogen) using the above-described procedures. PKCδ-TRPV1 coexpression was determined using a goat anti-PKCδ antibody (1:50, Santa Cruz) and a rabbit antibody directed against the TRPV1 C-terminus peptide (1:500) [34].

### Calcium imaging

Scratching behavior was evoked with pruritic stimuli applied to the back skin where site directed scratching occurs. We expanded our functional analyses of neuronal physiology to include both thoracic and lumbar DRG. Mice were euthanized rapidly by decapitation and DRG removed and acutely dissociated using previously described methods [71]. Briefly, DRG were incubated in 45U papain/L-cysteine in Hank’s buffered saline solution (HBSS) without Ca^2+^ or Mg^2+^ and with 10 mM HEPES for 20 minutes at 37°C and 5% CO_2_. Ganglia were then washed, followed by 20 minute incubation in 1.5 mg/ml collagenase in HBSS + HEPES. Ganglia were then triturated with fire-polished Pasteur pipettes, the dissociated cells were filtered through a 40 μm cell strainer, and were plated on poly-D-lysine and collagen-coated glass coverslips. Cells were incubated overnight at 37°C in 5% CO_2_ humidified air in culture medium (Neurobasal A with B27, pen/strep, 2 mM glutamax, 5% fetal bovine serum (Gibco)). All experiments were performed within 24 hours of plating.

Cells were incubated in 3 μg/ml Fura-2 AM (Molecular Probes) for 30 minutes and then incubated for 30 minutes in external solution (in mM): 130 NaCl, 5 KCl, 2 CaCl_2_, 1 MgCl_2_, 30 Glucose, 10 HEPES. For each recording, a coverslip was placed in a perfusion chamber and perfused with external solution at room temperature. Cells were viewed under an inverted microscope (Olympus Optical, Tokyo, Japan) and images were captured with a Hamamatsu Orca camera. SimplePCI Software was used to draw regions of interest (ROI) around Fura-loaded cells prior to recording. The ratio of fluorescence emission at an excitation wavelength of 357 and 380 nm was measured for each ROI. The experimental protocol consisted of a 2 minute baseline followed by 30 second bath application of histamine (100 μM in external solution), >8 minutes of external solution wash, 10 second application of capsaicin (200 nM), >8 minutes of external solution wash, and 10 seconds of KCl (50 mM) followed by wash (<2 minutes) to determine live neurons. A 10% or greater change from baseline 357 nm/380 nm ratio was considered a response to histamine. Capsaicin experiments were performed similarly except with 10 second capsaicin application (200nM). For experiments using PKCδ peptide inhibitor and scrambled peptide, cells were incubated in 100 μM peptide solution (dissolved in external solution) for 30 minutes prior to recording (δV1-1 peptide inhibitor (Myr-SFNSYELGSL-NH2), peptide inhibitor scramble (Myr-GLSFSEYLSN-NH2), Biomatik).

## Abbreviations

PKC: Protein kinase C
G_q_PCR: G_q_ Protein-coupled receptor
MRGPR: Mas-related gene protein coupled receptor
CQ: Chloroquine
KO: Knock-out
WT: Wild type
DRG: Dorsal root ganglion
SC: Spinal cord
PLC: Phospholipase C
PIP_2_: Phosphatidylinositol
IP_3_: Inositoltriphosphate
DAG: Diacylglycerol
H_1_R: Histamine receptor 1
ERK1/2: Extracellular signal regulated kinase 1/2
CGRP: Calcitonin gene-related peptide
IB4: Isolectin B4
Scr: Scramble
Inh: Inhibitor
PBS: Phosphate-buffered saline
HBSS: Hank’s buffered saline solution
IHC: Immunohistochemistry
TRPV1: Transient receptor potential vanilloid receptor 1
TRPA1: Transient receptor potential cation channel, subfamily A, member 1
PGE_2_: Prostaglandin E_2_
NGF: Nerve growth factor
IL-6: Interleukin-6.
